# VING: a software for visualization of deep sequencing signals

**DOI:** 10.1186/s13104-015-1404-5

**Published:** 2015-09-07

**Authors:** Marc Descrimes, Yousra Ben Zouari, Maxime Wery, Rachel Legendre, Daniel Gautheret, Antonin Morillon

**Affiliations:** ncRNA, Epigenetics and Genome Fluidity, Institut Curie, PSL Research University, CNRS UMR3244, Université Pierre et Marie Curie, 26 rue d’Ulm, 75248 Paris Cedex 05, France; Institute for Integrative Biology of the Cell, CNRS, CEA, Université Paris Sud, Bâtiment 400, 91405 Orsay Cedex, France

**Keywords:** NGS signal visualization, Strand-specificity, High-quality figure, R, Galaxy

## Abstract

**Background:**

Next generation sequencing (NGS) data treatment often requires mapping sequenced reads onto a reference genome for further analysis. Mapped data are commonly visualized using genome browsers. However, such software are not suited for a publication-ready and versatile representation of NGS data coverage, especially when multiple experiments are simultaneously treated.

**Results:**

We developed ‘VING’, a stand-alone R script that takes as input NGS mapping files and genome annotations to produce accurate snapshots of the NGS coverage signal for any specified genomic region. VING offers multiple viewing options, including strand-specific views and a special heatmap mode for representing multiple experiments in a single figure.

**Conclusions:**

VING produces high-quality figures for NGS data representation in a genome region of interest. It is available at http://vm-gb.curie.fr/ving/. We also developed a Galaxy wrapper, available in the Galaxy tool shed with installation and usage instructions.

## Findings

### Background

NGS is now widely used to study all aspects of gene expression from chromatin conformation (Hi-C) to protein-DNA binding (chromatin immunoprecipitation sequencing, ChIP-seq), transcription (native elongating transcript sequencing, NET-seq), RNA abundance (RNA-seq) and translation (ribosome profiling). A common step in most NGS approaches is the mapping of sequenced reads to a reference genome and analysis of the resulting signal. Multiple tools have been developed for quantitative analysis of NGS data. However, data visualization remains difficult because of the large quantity of information to display. Genome browsers such as Artemis [1], IGV [2] or Gbrowse [3] enable rapid navigation along the genome and coverage visualization, but are not fit for accurate, publication-quality image, neither for displaying multiple libraries. Alternatively, combinations of software such as BEDtools [4] and R or Matlab functions can produce customized plots, but require programming skills. Likewise, the Gviz R package [5], which enables customized display of a variety of genome annotation tracks, including NGS data, requires mastering the R environment and R objects. Here, we describe ‘VING’, an R package dedicated to the custom visualization of NGS data that can be easily launched using a single Unix command line, or within the Galaxy environment. VING introduces functionalities to handle data produced by the most recent NGS protocols, in a strand-specific manner. The code is optimized to enable a fast figure generation, even for the largest mapping files and genomes.

### VING components

VING produces snapshots of genomic regions from any set of mapping and annotation files, using a single command line. VING combines: loading of bam mapping files with optional user-provided normalization factors, loading of gff annotation file(s), plotting of signal and annotated genomic features.

#### Inputs

VING uses as input bam alignment files [6] and gff annotation files (description of the gff format can be found at http://www.sanger.ac.uk/resources/software/gff/). VING loads bam files using the Bioconductor package “Rsamtools”. Single-end or paired-end data are allowed and the library type can be specified as a parameter to assign reads to the proper strands. For paired-end data, each properly paired read is loaded as one single fragment. Users can also provide weights for normalization of each bam file. Annotation files are read by a custom function that only loads genomic features within coordinates defined by the users, enabling a faster operation. Users can also select the features to display.

#### Signal visualization

**Fig. 1:**
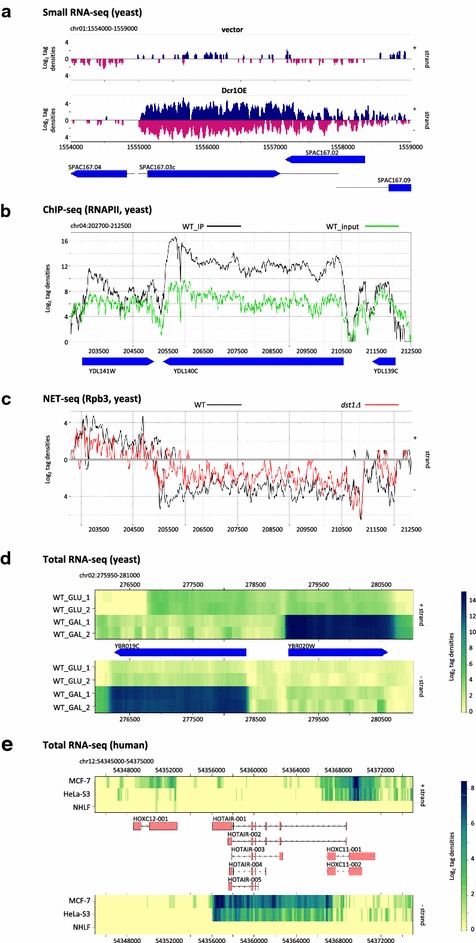
Examples of NGS signal visualization using VING. **a** Strand-specific “classic” visualization of 21–25 nucleotides small RNA densities along the *SPAC167.03c* locus in *rdp1*Δ *Schizosaccharomyces pombe* control cells (vector) or cells overexpressing Dcr1. Signal from each library is shown in a separate *panel*. Reads mapped on the + and − strands are shown on the *top* and *bottom* sides of the 0 *horizontal line*, respectively (additional representation in *different colors* optional). Annotated genomic features are represented as “*box*” (ORF) and “*line*” (mRNA). Original data described in [9]. The *Y axis* (log_2_ tag densities) shows the log_2_ of the number of reads (or pairs of reads in case of paired-end sequencing) at each position. **b** Unstranded “*line*” visualization of RNA Polymerase II ChIP-seq profile along the *YDL140C* (*RPO21*) locus in a wild-type strain of *Saccharomyces cerevisiae*. Signal intensity for each library is represented by a *different colored line* (IP, *black*; input, *green*). Strands are as in the “classic” view. Annotated ORF are represented as “*box*”. Original data described in [10]. *Y axis* see above. **c** Strand-specific “*line*” visualization of the NET-seq profile along the same region as B in wild-type (*black*) and *dst1*Δ (*red*) cells of *S. cerevisiae*. Original data described in [11]. *Y axis* see above. **d** Strand-specific “heatmap” visualization of the paired-end total RNA-seq signal along the *YBR019C*-*YBR020W* (*GAL10*-*GAL1*) locus in two biological replicates of *S. cerevisiae* wild-type cells grown in glucose- or shifted for 1 h in galactose-containing medium. *Distinct panels* are used for each strand. In *each panel*, *each lane* corresponds to one library. Signal intensities range from *white* (low) to *dark blue* (high). Annotated ORF are represented as “*box*”. Original data described in [12]. **e** Strand-specific “heatmap” visualization of the paired-end total RNA-seq signal along the *HOTA*IR locus in MCF-7, HeLa-S3 and NHLF cell lines. Annotated transcripts and exons are represented as “*arrow*” and “*rectangle*”. Original data from the ENCODE project described in [13]

The coverage signal (number of reads covering each nucleotide) is only computed for the requested genome area. Users may provide optional normalization factors for weighting each signal. These factors should be computed independently, either based on library sizes (RPM normalization) or using a dedicated package such as DESeq [7] or EdgeR [8]. The signal is plotted in a strand-specific manner using any of the three visualization modes: “classic” coverage plots using solid areas (each library in a distinct panel, Fig. 1a); “line” plots using lines of different colors and/or styles (one panel for all libraries, limited to 16 libraries, Fig. 1b, c); “heatmap” views based on a color-code to reveal high/low-density coverage regions (one panel for each strand, libraries shown as lanes in each of the two panels, no limitation of samples, Fig. 1d, e). Output files can be produced in high-resolution (300 dpi) tiff, jpeg, png or pdf format.

#### Annotation representation

Users can define a color and shape for each type of annotation feature (Fig. 1). Shapes include “box” (rectangle with an arrow at one side indicating the feature orientation), “rectangle” (plain rectangle), “arrow” (line with an arrow indicating the orientation) and “line” (straight line). VING automatically groups the different annotated features corresponding to the same ID such as untranslated regions (UTRs), exons and introns (or any other feature) from the same transcript, provided that these features are defined in the gff annotation file.

#### Performance

VING was tested on a variety of NGS data from different species, including yeast small RNA-seq (Fig. 1a), ChIP-seq (Fig. 1b), NET-seq (Fig. 1c), total RNA-seq (Fig. 1d), and human total RNA-seq data (Fig. 1e). Execution time depends on input files size. On an Intel Xeon 2,4 GHz processor with 32 Gb RAM, runtime ranged from 5 s and 2 min for the smaller (such as for Fig. 1a) and larger datasets (such as for Fig. 1d, e), respectively. Memory usage was under 500 Megabytes in all cases.

#### Usage

VING can be operated as a single command line. For graphical interface operation, we wrote a Galaxy wrapper enabling the users to input all parameters through the user-friendly Galaxy interface (available in the Galaxy Tool Shed: https://testtoolshed.g2.bx.psu.edu/view/rlegendre/ving).

## Conclusion

The VING program produces high-quality figures for NGS data representation in a genome region of interest. VING input and outputs have been rendered Galaxy-compatible so that automated coverage plots can be easily incorporated in Galaxy pipelines. The resulting, integrated view of a genome region is immediately suitable for figure production.

## Availability and requirements

Project name: VING.

Project home page: http://vm-gb.curie.fr/ving/.

Operating system(s): Linux. VING has also been successfully tested on MacOSX and Windows 7.

Programming language: R.

Other requirements: Bioconductor packages GenomicRanges and Rsamtools.

License: GNU GPL (version 3, 29 June 2007).

Any restrictions to use by non-academics: none.

## Availability of supporting data

Original raw data used in Fig. 1a, c–e were retrieved from the NCBI Gene Expression Omnibus, accession numbers GSE52535, GSE25107, GSE63444 and GSE26284, respectively. Original raw data used in Fig. 1b were retrieved from the NCBI Sequence Read Archive, accession number SRA030505. Truncated bam and gff files used for figure generation are provided on the VING website.

## Abbreviations

bam: binary alignment/map
ChIP-seq: chromatin immunoprecipitation sequencing
gff: general feature format
NET-seq: native elongating transcript sequencing
NGS: next generation sequencing
ORF: open reading frame
RNA-Seq: RNA sequencing
UTRs: UnTranslated regions
